# Taking it a step farther: acknowledging librarians’ systematic review work in the promotion or tenure process

**DOI:** 10.5195/jmla.2026.2202

**Published:** 2026-04-01

**Authors:** Rebecca Raszewski, Abigail Goben

**Affiliations:** 1 raszewr1@uic.edu, Associate Professor & Information Services & Liaison Librarian, Library for the Health Sciences, University of Illinois Chicago, Chicago, IL; 2 agoben@uic.edu, Professor & Data Management Librarian - University Library, and Data Policy Advisor - Office of the Vice Chancellor for Research, University of Illinois Chicago, Chicago, IL

**Keywords:** Systematic Reviews, Evidence Synthesis, Annual Review, Interdisciplinary, Promotion, Tenure

## Abstract

Librarians' contributions to systematic review projects receive inconsistent recognition within promotion or tenure processes. A review of thirty-six academic libraries' norms and procedures revealed only two that mentioned systematic reviews. Recognition and inclusion of systematic reviews and other evidence synthesis is further complicated by variance in recognition of interdisciplinary work. This commentary provides recommendations for academic library leadership to establish standards for documenting and evaluating systematic review work in annual reviews and promotion or tenure, explicitly recognizing the value of participation in interdisciplinary scholarship, inclusion of search strategies as a scholarly output, and providing guidance for the external review process. We close with a call to action for professional organizations to establish centralized guidelines to ensure the full recognition of librarianship and scholarly participation in systematic reviews.

Librarians have been recognized as important contributors to systematic reviews for over twenty years [1–3]. Over these last two decades, librarians have become regarded as key partners for this research methodology, with their expertise in search strategy development increasingly leading to co-authorship opportunities. This growth is an acknowledgement of the time health information professionals have devoted to building partnerships and collaborating with clinicians, faculty, researchers, and staff in other disciplines. While professional library associations and academic health sciences libraries now offer robust educational programs on the systematic review process and how to develop enterprise-level systematic review services, few libraries properly recognize the individual contributions their librarians make to the systematic review processes within their promotion, tenure, or annual reviews.

In this commentary, we are using the term systematic reviews as being synonymous with the term evidence synthesis, though we recognize that the latter also encompasses other kinds of review methodologies such as integrative, rapid, or scoping reviews. Additionally, while this commentary primarily focuses on the impact for librarians working in health sciences settings, systematic review work is becoming more commonplace for librarians across a variety of other disciplines.

Consulting or teaching about the systematic review process have become important aspects of most academic health sciences libraries. Librarians have developed competency frameworks [4–7], systematic reviews services [8–15], forms or systems for collecting information to develop protocols or documenting their work [16–18], and strategies for negotiating their involvement within systematic review development [19–21]. They have co-developed reporting standards such as PRISMA and validated search hedges [22,23]. Although the role of librarians and the credit they are given have been debated [24–26], librarians will continue to be involved in this process, whether as co-authors or consultants.

Health sciences library organizations such as the Medical Library Association (MLA) have acknowledged expert searching within professional competencies [27,28] and standards [29,30], recognizing this as often being the primary role of a librarian on a systematic review team. To support librarians’ development, MLA and other health information and non-profit organizations offer continuing education opportunities for librarians on conducting systematic reviews and gaining professional recognition for their participation [31–34]. Additionally, librarians who seek Academy of Health Information Professional (AHIP) certification can list the citations of published systematic reviews they have co-authored as part of establishing or maintaining their credential [35]. Yet it is unclear whether or how academic libraries value their librarians’ work on systematic reviews as part of the promotion process or in annual reviews.

The authors reviewed thirty-six United States academic libraries’ promotion or tenure norms where librarians are employed as faculty or have faculty status, including the authors’ own institution. The included institutions are prolific for their systematic review work or serve as regional libraries within the National Network of Libraries of Medicine. The norms were obtained from searches on libraries’ websites, interactions through libraries’ chat services, or email or ticket requests to individual librarians or departments. Systematic reviews were only mentioned in two of the thirty-six libraries’ promotion guidelines. The Spencer S. Eccles Health Sciences Library at the University of Utah mentions “novel systematic reviews” as an example under the creation of new knowledge category that also includes original research articles, scholarly monographs, presentations, and other kinds of research [36]. A related category, novel synthesis of existing knowledge, would give librarians credit for literature reviews or “review that proposes new conceptualizations of existing evidence.” The University of Florida mentions systematic reviews and meta-analyses with peer reviewed articles as “indictors of distinction” for promotion [37]. The lack of acknowledgement within promotion guidelines suggests that academic and health sciences libraries have not recognized librarians’ critical role in the systematic review process and how the time-intensive nature of these projects should be viewed within annual reviews and promotion processes.

## RECOMMENDATIONS

Systematic review work must be included in annual reviews and promotion and tenure norms so that this labor and intellectual contribution is documented and recognized. Below we outline specific suggestions for what should be tracked to provide appropriate recognition for this work.

### Annual Reviews

As a part of the annual review process, librarians who are co-authors must receive scholarly credit for any systematic reviews that are in process, submitted for publication, or have been published. Additionally, librarians must document instances when they completed a search, but a co-authored publication did not result. They should also include the number of consultations conducted as well as the amount of time spent on review-related tasks like translating searches into databases, citation tracing, or writing the methods section. To facilitate and standardize this across librarians at a given institution, an internal tracking system should be created or adopted to support librarians documenting their systematic review work so that they can quickly and consistently pull reports. A robust tracking system will ensure that the librarians’ impact is documented for library leadership and for reports out to central campus administration. Additionally, these internal reports should be used to assess workloads and evaluate whether the quantity of systematic review requests is sustainable relative to current staffing levels [38]. It will also allow the identification and disruption of any trends where librarians are being inappropriately excluded from co-authorship when partnering with specific colleges or departments. As systematic reviews often require significant effort by the librarian at the beginning of the project, which may or may not see follow-through by the rest of the team, we recommend a written Memorandum of Understanding [39,40] by the team prior to project initiation, and to review ICMJE [41] rules for co-authorship and document team expectations towards publication.

If there is a disconnect between how librarians value their systematic review work versus their administrators or peers, then library administration or senior librarians need to initiate discussions to resolve this issue and ensure evaluation consistency. This is especially critical at institutions where librarians are expected to produce original scholarship as a criterion for evaluation. Supervisors and mentors will need to establish, support, and reinforce boundaries on their junior colleagues’ involvement in systematic review work so they can develop their independent scholarship in addition to their systematic review contributions. This will likely need to continue to be handled on an individualized level, but library supervisors and leadership should recognize this labor and critically appraise the efforts being allocated to this work, especially if follow-through to publication is not consistently achieved.

### Promotion or Tenure

As systematic reviews are now a recognized form of scholarly activity across most disciplines, librarians must revise promotion and tenure norms and other promotion standards to specify how their colleagues’ will receive credit for their systematic review work for scholarship and/or librarianship requirements. This should be supplemented with clear guidance for departmental, campus, and external reviewers, to reduce confusion and inconsistent evaluation.

Systematic reviews are typically conducted on topics that are outside of the Library and Information Science (LIS) discipline, which at some institutions may not be assigned to the librarians’ primary area of scholarship but to interdisciplinary work or counted as part of their librarianship. Even when evaluated as scholarship, one 2016 study did not find consensus as to whether systematic reviews were more or less important than LIS scholarship [26]. In the years since, this has not changed. During our review of libraries’ norms, only 11 of the 36 libraries’ promotion guidelines included text that valued interdisciplinary work or publications the same as LIS publications. One library’s norms mentioned they “may be an indicator of distinction”, while another library valued interdisciplinary work less. Where in alignment with campus standards, systematic reviews are likely to best fit into an interdisciplinary statement of scholarship.

Librarians in faculty positions may be expected to develop their own independent research agenda in addition to their contributions to systematic reviews. Participating as the librarian member of a systematic review team may not be considered a demonstration of unique scholarship nor an advance in the knowledgebase of librarianship. Norms should identify expectations for librarians required to pursue their own independent research, with appropriate support, with the addition of recognition and credit for participation in interdisciplinary research including systematic reviews. Clear documentation and expectations in this respect will allow librarians to balance their commitment of effort between their own scholarly-led efforts and serving as part of an interdisciplinary team on a systematic review. Systematic reviews where the librarian serves as the lead author or where the topic is directly connected to their scholarship, such as a review on nursing information literacy interventions, would fall under the librarian’s own scholarly agenda. Library norms should indicate if a number of articles is required, and what balance will be accepted between a librarian’s personal scholarship and their interdisciplinary scholarship.

### Search Strategies

Librarians should also consider including the search strategies or search hedges they have developed in their annual reviews or promotion packets as a supplemental form of scholarship If the search strategies are not included within the affiliated reviews, they should create an affiliated record in PROSPERO, place them in their institutional repository or a resource like Open Science Framework (OSF), or plac them on a LibGuide or similar resource to allow peer discovery and reuse. Some Canadian libraries have begun posting their search strategies in their institutional data repository to better highlight and control their usage [42–46]. SearchRxiv, a search strategy archive that allows searches to be shared and re-used, is another opportunity for librarians to highlight their search strategies [47]. Additionally, Haddaway et al’s 2022 article includes recommendations for a data structure that can be used to report reproducible search strategies [48]. Librarians could also include the number of times a search strategy or hedge has been viewed or even cited in their promotion packets. They should also ensure that they are giving proper credit to other librarians by citing their publications or search strategies if they have used them or integrated them into their search strategies.

### Guidance for External Review

As libraries have a variety of norms and expectations for promotion, it is incumbent that leadership and senior librarians create clear and standardized guidance that assists external reviewers in understanding how the library values systematic review work: if these reviews count towards librarianship or scholarship, and the expected balance between librarian-led work versus collaborative work on interdisciplinary projects. Without this guidance, reviewers from other institutions may be confused as to why systematic review work is not being fully reflected within candidate’s statements, CV, or other dossier materials.

One technique to facilitate this would be the creation of an annotated bibliography where the candidate describes their role in each of their scholarly outputs. For systematic reviews this could include protocols, the specific tasks performed (searching, deduplication, title/abstract screening, etc.), and/or the total amount of time spent on each phase of the review. Additionally, we recommend advocating for a separate interdisciplinary statement in addition to the traditional teaching, scholarship, and service statements for all librarians to address their engagement across disciplines.

As part of our dossier for promotion and tenure cases, University of Illinois Chicago (UIC) University Library gives library faculty the option to write and include an interdisciplinary statement. Most of the health sciences library faculty write an interdisciplinary statement to highlight their systematic reviews and other interdisciplinary scholarship, their service work at other colleges such as curriculum or search committees, or their service work at their hospital such as hospital-wide or nursing councils. When the interdisciplinary statement has included scholarship, the candidate has the option of sharing it with external reviewers to more fully document how their systematic review and other interdisciplinary work fits in with their scholarship. UIC Library also encourages faculty to annotate their citations within their CVs and dossiers so external reviewers and the campus-wide Promotion and Tenure Committee can better understand the librarian’s role or contributions [49].

## CALL TO ACTION FOR PROFESSIONAL ASSOCIATIONS

To reduce the replication efforts of documenting systematic review work across organizations, associations such as MLA or the Association of Academic Health Sciences Libraries (AAHSL) should develop and provide recommendations to their members on how to document their contributions to systematic reviews for annual reviews and promotion. A taxonomy for systematic review work like CRediT could be developed that would define the librarians’ contributions to various phases of the systematic review process [50]. The health sciences librarianship literature has a few examples of articles that cover librarians’ roles in the systematic review process [51–53]. It could also be used by librarians who work on systematic reviews that are never completed, or whose role as search consultants may only be documented as an acknowledgement in published systematic reviews for their work [25,26]. An example of this is present in the Cochrane Collaboration Contribution of the Authors section [54]. However, as many systematic reviews are not completed or published, this will be an incomplete representation of librarians’ efforts. Similarly, due to the time frame for publication, some librarians may need documentation prior to submission or publication of their work on the reviews.

Librarians lay the foundation for the systematic review process. The review cannot proceed until the database searches are developed, translated, and finalized; the final citations are downloaded from databases; and duplicate citations are removed. Within our own institutions, we need to better demonstrate our contributions to fully capture our work within the systematic review process. We further need to discuss as a discipline how we are valuing the contribution of our expertise in systematic reviews not only to librarianship but beyond. Otherwise, we and our workplaces are failing ourselves by potentially undervaluing this scholarly labor or presenting an incomplete view of our work.
